# Measurement of Family-centered care perception and parental stress in a neonatal unit**^1^**

**DOI:** 10.1590/1518-8345.0710.2753

**Published:** 2016-08-08

**Authors:** Flávia Simphronio Balbino, Maria Magda Ferreira Gomes Balieiro, Myriam Aparecida Mandetta

**Affiliations:** 2 RN, PhD, Departamento de Enfermagem Pediátrica, Escola Paulista de Enfermagem, Universidade Federal de São Paulo, São Paulo, SP, Brazil.; 3 PhD, Adjunct Professor, Departamento de Enfermagem Pediátrica, Escola Paulista de Enfermagem, Universidade Federal de São Paulo, São Paulo, SP, Brazil.; 4 PhD, Associate Professor, Departamento de Enfermagem Pediátrica, Escola Paulista de Enfermagem, Universidade Federal de São Paulo, São Paulo, SP, Brazil.

**Keywords:** Interventions Studies, Family, Health Personnel, Neonatal Nursing, Neonatal Intensive Care Unit

## Abstract

**Objective:**

to evaluate the effects of the implementation of the Patient and Family-Centered Care Model on parents and healthcare perceptions and parental stress.

**Method:**

a quasi-experimental study developed in a neonatal unit of a university hospital in the municipality of São Paulo, Brazil, with the implementation of this model of care. Data collection were performed by two sample groups, one using non-equivalent groups of parents, and another using equivalent groups of healthcare professionals. The instruments Perceptions of Family-Centered Care-Parent Brazilian Version, Perceptions of Family-Centered Care-Staff Brazilian Version and Parental Stress Scale: Neonatal Intensive Care Unit, were applied to 132 parents of newborns hospitalized and to 57 professionals.

**Results:**

there was a statistically significant improvement in the perceptions of the parents in most items assessed (p ≤0,05) and for the staff in relation to the family welcome in the neonatal unit (p = 0.041) and to the comprehension of the family's experience with the infant´s hospitalization (p = 0,050). There was a reduction in the average scores of parental stress, with a greater decrease in the Alteration in Parental Role from 4,2 to 3,8 (p = 0,048).

**Conclusion:**

the interventions improved the perceptions of parents and healthcare team related to patient and family-centered care and contributed to reducing parental stress.

## Introduction

In recent decades, authors have been widely discussing the importance of family participation in patient care, showing the need to caring for the family in the context of hospitalization, with health team support, based on a model of care that may bring physical and emotional benefits for both^1^ ^-^ ^2^.

The Patient and Family-Centered Care (PFCC) Model has been proposed as an innovative approach to the planning, provision and health evaluation, conducted mutually by the partnership between health care providers, patients and families. It can be applied to patients of all ages and practiced in any health facility^2^ ^-^ ^3^.

In the neonatal context, studies show that the PFCC is becoming the standard of care in the world, where the family is understood as a primary source of strength and support of the newborn^4^ ^-^ ^5^. This perspective of care incorporates concepts such as unrestricted access to the child, respect, information, choice, service flexibility, autonomy of the involved subjects, cooperation and support at all levels of service provision^5^.

The benefits that have been demonstrated on the PFCC refer to the improvement of health and well-being of the newborn and his family, that translate into: greater satisfaction^6^, efficiency, access and communication^7^; decreased newborn's hospital stay and readmissions^8^; reducing parental stress and increase the self-confidence of parents after discharge^9^; greater adherence to "kangaroo" care and developmental care^10^; strengthening the bond between newborn and family, increased breastfeeding rate with better mental health outcomes in the long term and greater satisfaction of the health team in the care^4^. However, these benefits do not show strong evidence for recommendations, justifying the need for clinical studies. 

In Brazilian neonatal units there is a constant demand of the parents to participate in the care of their children, combined with relational difficulties faced in interactions with the multidisciplinary team, revealing that the PFCC philosophy is still not a reality in most of these contexts^11^. 

Fostering organizational cultural change requires reframing of beliefs, values and attitudes of the involved professionals^12^. This is a slower process in critical care environments, because many professionals who work in this context are attracted by hard technology (understood as devices), and the service dynamics is focused on the disease and not on the soft technology of relationships^3^. 

Thus it is necessary that professionals broaden their focus of care, from an approach focused on illness to one that includes family, covering the essential elements of the PFCC; changing attitudes, beliefs and professional values that restrict access and participation of the family in this environment, acknowledging for the vulnerability and suffering of the family, as well as its potential and its central and permanent role in the child's life^2^ ^,^ ^5^.

It has being challenged whether the implementation of the PFCC Model in the neonatal unit can contribute to a change in the culture of professionals, evidenced by more positive perception of the families and health professionals about the core elements of this model of care and also whether it contributes to the reduction of parental stress.

The aim of this study was to evaluate the effects of the implementation of the Patient and Family-Centered Care Model in the perceptions of parents and health professionals and in parental stress.

## Methods

This is a quasi-experimental study with two sample groups, one using non-equivalent groups of parents, and another using equivalent groups of healthcare professionals to evaluate the effects of the implementation of PFCC Model.

The study was performed at a neonatal unit of a university hospital in the municipality of Sao Paulo. This unit is a reference center for the care of high risk and malformed newborns and has specialized professionals and technology to meet the needs of this specific population. 

The parents' sample consisted of 132 representatives of families being them the father, the mother or both, divided into independent groups, 66 in the pre-intervention and 66 in the post-intervention phase. It is noteworthy that the non-equivalent sample in the group of parents is justified by the turnover of patients in the neonatal unit during the data collection period, as the variation was of five months.

From a population of 77 professionals, the sample encompassed 57 members of the healthcare team.

The inclusion criteria adopted for family representatives was being a parent of a hospitalized newborn in the last 72 hours or more. For healthcare team, the criteria were: higher education graduates and to be developing care activities for at least one (1) year in the unit. 

Exclusion criteria were to be parents of a newborn in the end of life care and for professional to be on vacation or out of work during the period of data collection.

The project met the principles of Resolution No. 466/12. In this way, the program began with the approval of the Ethics Committee of the institution, case No. 042/11.

### Intervention: Implementation Program of the Patient and Family-Centered Care Model in the Neonatal Unit (IPPFCCM-NU).

The IPPFCCM-NU was carried out to lead the change in organizational culture, promoting the inclusion of the family in this context. For its implementation, the phases of the Theory of Planned Change^13^ were used.

The first, called *unfreezing*, was conducted through an agreement upon the proposed change with the coordinators of the neonatal unit, followed by a conference involving all professionals of the unit (two months).

The second phase, the *transition to the new*, consisted of the development of a guide of best practices with families in the neonatal unit, containing the philosophy and guidelines related to the actions: (a) opening of the unit to parents within 24 hours; (b) entry of other family members (brothers, grandparents and significant people for parents); (c) participation of parents in care; (d) offering to share information; (e) family embracement in situations of loss and grief; (f) mediation of conflicts between parents and staff; (g) participation of parents in decision-making process concerning newborn care. Additionally there were training activities for the multidisciplinary team, for embracing the family in a neonatal unit considering the assumptions of the PFCC philosophy that advocates individualized care with respect and appreciation from the perspective of the family (three months).

The latter phase, called *refreezing phase* was carried out in two stages: (1) internalization of the new, when the guide of best practices with the family was put in place from July 2013; and (2) evaluation of the program by application of measuring instruments, after three months of its implementation.

### Data collection

For assessing the effect of the intervention, the perception of the family and staff about the PFCC and the level of parental stress, were defined as study variables.

The perception of the PFCC was evaluated through the application of two measuring instruments, called Perceptions of Family Centered Care -Parents (PFCC-P), Brazilian version and Perceptions of Family Centered Care - Staff (PFCC- S) Brazilian version^14^ ^-^ ^15^. They were composed by 20 questions divided into three domains: respect, collaboration and support. The domain *respect* includes six items about recognizing the family rights in the hospital. The domain *collaboration* reflects the recognition of the role of parents in a partnership for theirs children care, and comprises nine items. The domain *support* includes five items related to the way health team professionals offer support to the family. The answers to each question range in a Likert scale with four options: never, sometimes, often and always; with scores ranging from 0 to 3^14^. 

The level of parental stress was measured by the Parental Stress Scale: Neonatal Intensive Care Unit (PSS: NICU) adapted to the Brazilian Portuguese^16^, consisting of 26 items divided into three subscales "sounds and sights", "baby looks and behaves" and "alteration in parental role". Parents pointed on a Likert scale with scores between 1 and 5, in which point they experienced stress in the scale items. The score "1" refers not at all stressful, "2" a little stressful, "3" moderately stressful, "4" very stressful and "5" extremely stressful ^(^ ^16^.

The evaluation of PSS:NICU instrument responses can be made by Metric 1 or Stress Occurrence Level corresponding to the level of stress in which the situation happens; and the Metric 2 or Overall Stress Level, referring to the general level of environmental stress^17^.

The variables related to parents include socio-demographic profile, distance and time to reach the hospital and the experience with hospitalization of children and social support. Regarding the variables related to team members, they were gender, age, educational level, unit where they work and profession; and neonatal variables were length of stay, age and diagnosis.

Data were collected pre-intervention, and then three months post-intervention (implementation of IPPFCCM-NU).

### Data analysis

The analysis of categorical variables was performed using absolute frequencies (n) and relative frequencies (%), and the numerical ones, by mean, median, quartiles and standard deviation. To analyze the perception of the PFCC by parents and identification of change in parental stress level in the pre- and post-intervention, the statistical technique *Mann-Whitney* test was used, because it dealt with non-equivalent groups. To analyze the perception of healthcare team pre and post-intervention, the *Wilcoxon* test was used, because it is a comparison among the participants themselves. The associations between demographic variables and the perception of parents and health professionals about the items of the PFCC-P and PFCC-S, Brazilian version were analyzed using *Fisher's exact test*.

## Results

Parents have similar characteristics in the pre-intervention and post-intervention, as shown in Table 1. It is noteworthy the predominance of the female gender and age group between 31 to 45 years, with incomplete high school educational level. The parents spend an average of 1 to 2 hours traveling to the hospital. These are families that have a child under parental care and have never had experience with other child hospitalization in the family group.

**Table 1:** Socio-demographic profile of parents of newborns admitted to the neonatal unit. Sao Paulo, SP, Brazil, 2013 (N = 132)

Variables		Pre-intervention		Post-intervention	
		n	%	n	%
Gender					
	Feminine	40	60,6	44	66,7
	Masculine	26	39,4	22	33,3
Age groups					
	16 a 20 years	8	12,1	7	10,6
	21 a 25 years	10	15,2	15	22,7
	26 a 30 years	16	24,2	16	24,2
	31 a 45 years	32	48,5	26	39,4
	46 a 50 years	0	0,0	2	3,0
Educational Level					
	Elementary (not complete)	11	16,7	8	12,1
	Elementary (complete)	12	18,2	6	9,1
	High School (incomplete)	21	31,8	30	45,5
	High School (complete)	6	9,1	8	12,1
	Technical School	2	3,0	1	1,5
	Higher Education (incomplete)	4	6,1	7	10,6
	Higher Education (complete)	9	13,6	6	9,1
	Post Graduate	1	1,5	0	0,0
Distance home-hospital					
	Near	6	9,1	3	4,5
	Halfway	4	6,1	3	4,5
	Distant	56	84,8	60	90,9
Time to arrive to the Hospital					
	Less than 30 minutes	8	12,1	6	9,1
	30 minutes to 1hour	23	34,8	15	22,7
	1 to 2 hours	35	53,0	37	56,1
	More than 2 hours	0	0,0	1	1,5
Number of children under care					
	One	32	48,5	25	37,9
	Two	19	28,8	26	39,4
	Three	8	12,1	9	13,6
	Four	5	7,6	6	9,1
	More than four	2	3,0	0	0,0
Support for child care					
	Yes	43	65,2	44	66,7
	No	23	34,8	22	33,3
Previous hospitalization experience					
	Yes	16	24,2	11	16,7
	No	50	75,8	55	83,3

As for the professionals, most are female, mainly physician and nurses, both with specialties, in the age groups between 31 and 45 years and median working time with newborns of 7 years (Table 2).

**Table 2:** Socio-demographic profile of professional health staff of the neonatal unit. Sao Paulo, SP, Brazil, 2013 (N = 57)

Variables		Pre-intervention / Post intervention	
		n	%
Gender			
	Feminine	52	91,0
	Masculine	5	8,7
Age Groups			
	25 years or less	3	5,2
	26 a 30 years	21	36,8
	31 a 45 years	26	45,6
	46 a 50 years	3	5,2
	51 a 55 years	2	3,5
Educational Level			
	Post Graduate	50	87,7
	Higher Education	7	12,2
Time working with newborns (years)			
	Minimum	1	
	Median	7	
	Maximum	25	
Profession			
	Physician	33	57,8
	Nurse	14	24,5
	Physiotherapist	6	10,5
	Speech Therapist	2	3,5
	Psychologist	1	1,7
	Social Worker	1	1,7
Function			
	Resident	12	21,0
	Care/By day	29	50,8
	Work in Shifts	16	28,0
Title			
	Specialist	37	64,9
	Master's Degree	10	17,5
	PhD	3	5,2
	No title	7	12,2

The total number of newborns whose parents participated in the study was 98, of them 47 in the pre-intervention and 51 in the post-intervention. Among these newborns there were four twins in the pre-intervention phase and two in the post-intervention phase. The most common diagnosis was prematurity, of them 53,2% in the pre-intervention phase and 49,0% in the post-intervention phase, followed by congenital anomalies with 38,3% in the pre-intervention and 29,8% in the post-intervention phase. At the time of data collection in the pre-intervention phase, the median length of stay of the newborn was 10 days, with a minimum of 3 and maximum of 120 days; and in the post-intervention phase it was 14 days, with a minimum of 3 and maximum of 180 days.

**Figure 1:**
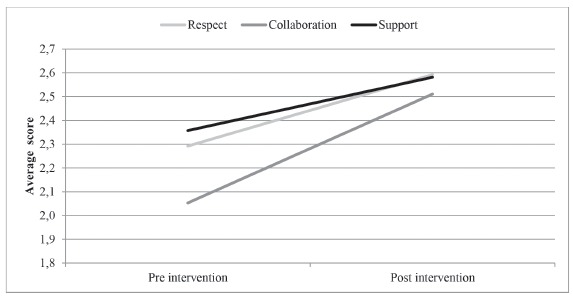
Effect of intervention in average scores of parental response by dominions in the Perception of Family Centered Care -Parents (PFCC-P) Brazilian version. Sao Paulo, SP, Brazil, 2013.

The answers regarding the perception of parents on the PFCC with the intervention showed an increase in average scores for all areas of the PFCC-P and the PFCC-S, Brazilian version (Figure 1), but with a greater increase in positive responses to the domain Collaboration (mean score of 2.05 value - pre-intervention, going to 2.51 - post-intervention), which include questions related to the preparation of discharge, sources of support, inclusion of the family in decision making and care, shared information, identification of the professional responsible for the care of the child, understanding of instructions received and sense of relief with the information received. 

By analyzing the IPPFCCM-NU intervention effect on the parents' perception post-intervention, it was found that the score in the PCCF-P was statistically higher compared to pre-intervention time for Respect domain in questions: 3. *I am able to be with my child during procedures* (p <0,001); and 5. *I feel like a visitor (rather than a parent)when I come to the hospital* (p <0,001). In the domain Collaboration in the questions 7: *I feel prepared for discharge / referral to other community services after my child*´s *discharge* (p = 0,013*); I know who to call after I get home if I need help or reassurance (p = 0,002); 10. When decisions are made about my child's care the staff include me* (p = 0,005); *11. I am taught what I need to know about my child's care* (p = 0,006); 12*. I know the name of the doctor in charge of my child's care* (p = 0,014); 13. *I understand the written material that has been given to me* (p = 0,049), 14. *My family is included in my child*´s *care* (p = 0,039); and 15. *I feel overwhelmed by the information given to me about my child* (p <0,001). In the Support domain on these questions: 18. *I get to see the same staff* (p = 0,032); and 19. *The staff knows who my support people are* (p = 0,008). For other questions was not possible to verify changes in score when comparing the moments before and after the intervention. 

**Figure 2:**
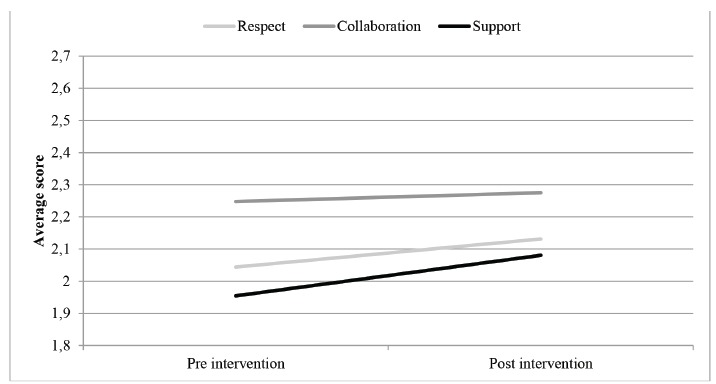
Effect of intervention in average score of the answers of health team staff by domains of the Perceptions of Family Centered Care-Staff (PFCC-S), Brazilian Version. Sao Paulo, Brazil, 2013.

Regarding the responses of the health team staff, a more positive perception was identified in the domains Respect (going from average score of 2.04 to 2.13) and Support (from 1.95 to 2.08) with the intervention (Figure 2). Thus, it was found by statistical analysis that there was a significant difference in respect to the Respect domain for the parents reception on arrival at the hospital (p = 0,041) and in the Support domain, related to the understanding attitude the team had, regarding the experience of parents (p = 0,05).

After the intervention, there was 30% improvement in the perception of the health team professionals regarding the PFCC related to the participation of the extended family, the presence of parents during procedures, the inclusion of the family in child care and the knowledge of the parents' support network. 

Regarding the level of occurrence of parental stress (Metric 1) was evident that was statistically lower (less stressful), compared to the time before the intervention in the subscale Alterations in Parental Role, the items related to: baby separation (p = 0,042); could not hold the baby (p = 0,027); and feel unable to help your baby (p = 0,010). But it was more stressful on questions related to the Baby Look and Behavior, in the item referring to the baby's size (p = 0,038).

Considering the environmental stress level (Metric 2), parental stress before intervention was statistically equal to the moment after the intervention for almost all questions in the PSS:NICU scale, with the exception of questions relating to the Alterations in Parental Role in items related, to not being able to hold the baby when I want to (p = 0,030) and feeling unable to help their baby (p = 0,003) demonstrating that there was a reduction of stress related with the intervention carried out.

**Figure 3:**
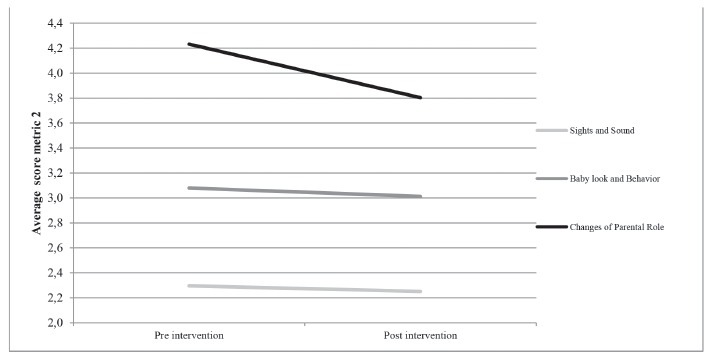
Average score of Parental Stress by metric 2. Sao Paulo, SP, Brazil, 2013

The effect of IPPFCCM-NU presented a decrease in mean scores of parental stress in the domains of PSS:NICU scale, showing greater decline in the domain Alterations in Parental Role (4,2 to 3,8), representing a decrease of stress going from very stressful in the pre-intervention phase to moderately stressful in the post-intervention phase (Figure 3).

## Discussion

The IPPFCCM-NU contributed to statistically significant changes in the more positive perception of parents at the three domains of the instrument Perceptions of Family Care Centered-Parents (PFCC-P), Brazilian version: Respect, Collaboration and Support, corroborating what was evidenced by the authors of the instruments^17^ , i.e., that the parents also had significantly higher scores in all areas. Similar results were observed in a cohort study on the effectiveness of an integrated care program with family, involving the readiness of the family to take care of the newborn after being discharged. The authors identified improvement in parent-healthcare team relationship caused by increased collaboration between staff-family^4^.

Studies^18^ ^-^ ^22^ ^)^ related to family-centered care show that nurses have knowledge about the assumptions underlying this model of care, but also state that this knowledge is not yet fully incorporated into their practice^18^ ^-^ ^20^; that there are still difficulties in its implementation, such as scarce interprofessional collaboration, lack of continuing education programs addressing this issue^21^ and structural barriers in the health system for collaborative practice between parents and professionals, as recommended by the PCCF^22^.

Although there was an improvement of 30% in the perception of the health team professionals regarding the PCCF, the team still perceives a resistance for the presence of others than parents, grandparents and siblings. Resistances are not calculated or strategically planned, they are simply defensive reactions that may become other forms of beliefs^21^.

When establishing new knowledge to newborn care practice, there are perceivable forces in play, with professionals advocating for old behaviors such as isolation of the newborn and care focused on the disease, while others struggle for new practices, such as the opening the unit for the family, encouraging bonding^21^.

In the view of neonatal nurses there is greater need for readiness to implement this model of care in practice, through continuing education, with guidance and ongoing support of the institution^22^. However, it can be considered that the intervention as performed helped to trigger the process of change in the organizational culture, reflected by increased awareness of the multidisciplinary team regarding the embracement of parents in the unit and for the understanding of their experiences. The positive perception of parents reinforces this statement because there was an increase of most of post-intervention mean scores in all domains of PFCC-S, Brazilian version, with greater emphasis on collaboration.

With the intervention, parental stress measured in the Alterations in Parental Role subscale still remained in moderately stressful levels, due to the children removal, the restriction on the handling and the helplessness of parents in helping them. These data show that parental stress is multifactorial and that interventions that empower parents help in the construction of parenthood in a public space, helping to minimize their vulnerability^23^, defined by the loss of power to protect their children.

Reviews of intervention programs involving the family, in neonatal units, identify decrease the stress level of parents in the post-intervention period^24^; reducing maternal anxiety, through a collaborative care^25^; increased satisfaction of mothers with the care provided, and exacerbated parental feelings of well-being and increases parental ability to care for their babies^4^ ^,^ ^25^. 

The results of this study reflect an initial assessment in the short term, in which the parents show more positive answers than the team, which may indicate that this care philosophy is being incorporated gradually, suggesting that the team needs a process of continuing education, in order to occur a strong change in the culture of these professionals.

Although the results point to an improvement in the perception of parents and health practitioners of the team with the IPPFCCM-NU, the study was limited to two measures (parental stress and perceptions of the PFCC by parents and healthcare team professionals).

It is thus reinforced the importance of continuing the implementation, improving interventions and conducting prospective studies correlating the PFCC with neonatal variables, such as weight gain of the newborn, length of stay, exclusive breastfeeding at hospital discharge and evaluation of post child development after discharge. It is recommended also to correlate the PFCC with parents' variables, such as satisfaction, anxiety and autonomy; and team variables such as satisfaction with the care provided, level of self-esteem, among other relevant aspects.

## Conclusion

In this study it was found that there was significant improvement in the perception of parents in relation to Patient and Family Centered Care in the dimensions respect, collaboration and support in the post-intervention phase. Parents responded more positively about family-centered care that health team members before and after the intervention.

The health team professionals after the intervention showed a probability above 30% in the improvement of positive responses on the participation of the extended family, the presence of parents during procedures, family inclusion in child care and knowledge of the parents' support network. In addition there was a statistically significant improvement in the perception of the health team professionals in the post-intervention phase, in relation to family acceptance and greater understanding of the experience of parents living with a hospitalized child in the neonatal unit.

Parental stress was reduced after the intervention and it was statistically significant lower in the subscale Alterations in Parental Role, regarding the items: "I can not hold my baby when I want" and "feeling helpless e about how to help my baby during this time."

It was concluded that the IPPFCCM-NU interventions have improved the perception of parents and health team on the PFCC and contributed to reducing parental stress.
